# Cobalt–Imidazole Complexes: Effect of Anion Nature on Thermochemical Properties

**DOI:** 10.3390/ma17122911

**Published:** 2024-06-14

**Authors:** Olga V. Netskina, Dmitry A. Sukhorukov, Kirill A. Dmitruk, Svetlana A. Mukha, Igor P. Prosvirin, Alena A. Pochtar, Olga A. Bulavchenko, Alexander A. Paletsky, Andrey G. Shmakov, Alexey P. Suknev, Oxana V. Komova

**Affiliations:** 1 Boreskov Institute of Catalysis SB RAS, Pr. Akademika Lavrentieva 5, 630090 Novosibirsk, Russia; sukhorukov@catalysis.ru (D.A.S.); k.dmitruk@g.nsu.ru (K.A.D.); msa@catalysis.ru (S.A.M.); prosvirin@catalysis.ru (I.P.P.); po4tar@catalysis.ru (A.A.P.); obulavchenko@catalysis.ru (O.A.B.); suknev@catalysis.ru (A.P.S.); komova@catalysis.ru (O.V.K.); 2 Department of Natural Sciences, Novosibirsk State University, 1 Pirogova Str., 630090 Novosibirsk, Russia; 3 Voevodsky Institute of Chemical Kinetics and Combustion SB RAS, 3 Institutskaya Str., 630090 Novosibirsk, Russia; paletsky@kinetics.nsc.ru (A.A.P.); shmakov@kinetics.nsc.ru (A.G.S.)

**Keywords:** gas-generation composition, cobalt complex, solvent-free synthesis, imidazole, high-energy ligand, oxidizing anion, cobalt oxide

## Abstract

A solvent-free method was proposed for the synthesis of hexaimidazolecobalt(II) nitrate and perchlorate complexes—[Co(C_3_H_4_N_2_)_6_](NO_3_)_2_ and [Co(C_3_H_4_N_2_)_6_](ClO_4_)_2_—by adding cobalt salts to melted imidazole. The composition, charge state of the metal, and the structure of the resulting complexes were confirmed by elemental analysis, XPS, IR spectroscopy, and XRD. The study of the thermochemical properties of the synthesized complexes showed that [Co(C_3_H_4_N_2_)_6_](NO_3_)_2_ and [Co(C_3_H_4_N_2_)_6_](ClO_4_)_2_ are thermally stable up to 150 and 170 °C, respectively. When the critical temperature of thermal decomposition is reached, oxidative two-stage gasification is observed. In this case, the organic component of the [Co(C_3_H_4_N_2_)_6_](NO_3_)_2_ complex undergoes almost complete gasification to form Co_3_O_4_ with a slight admixture of CoO, which makes it attractive as a component of gas-generation compositions, like airbags.

## 1. Introduction

To obtain a large volume of gaseous products within a short time, gas-generation compositions are used. They are in demand in fire extinguishing [1], autonomous systems for lifting sunken objects [2], car airbags [3], and systems for intensifying oil production, especially highly viscous types [4]. The required components of gas-generation compositions are an oxidizer, a binder, and an energy additive (fuel). However, they can interact with each other during storage, leading to degradation in their performance. From this point of view, complex compounds of transition metals, which contain high-energy ligands and oxidizing anions, are more attractive. In addition, the metal can act as a catalyst, accelerating the thermal decomposition of the complex.

Currently, a significant number of metal–organic complexes of transition metals have been synthesized, but to be used in gas generators, they should meet strict requirements, including thermal stability, complete gasification, and resistance to external influences, and should not be transformed under the action of atmospheric oxygen or water vapor. Moreover, their synthesis should be simple, low-waste, and without the use of expensive equipment. The simplest way is the synthesis of metal–organic compounds without solvents by mixing solid metal salts with good chelating ligands [5], for example, ball mill use [6]. However, it is difficult to achieve phase homogeneity, which reduces the yield of metal–organic compounds and requires further purification of the target product from the impurities of the reagents. To obtain metal–organic complexes from thermally stable compounds, it was proposed to melt their precursors in a stoichiometric ratio [7], which was developed in our research works [8,9]. This approach is especially interesting for complex compounds, which should be synthesized in well-dried solvents to prevent the complete or partial hydrolysis of the reagents or products of their interaction.

In this work, we investigated hexaimidazolecobalt(II) nitrate and perchlorate complexes prepared by solvent-free dry-melt synthesis. The purpose of our study was to establish the dependence of their thermochemical properties and the composition of gasification products on anion nature.

## 2. Results and Discussion

### 2.1. Study of the Cobalt Complexes

Hexaimidazolecobalt(II) nitrate and perchlorate complex compounds were synthesized by adding cobalt salts to the melted imidazole, which significantly reduced the precursors’ synthesis time. The elemental analysis of the acquired products showed that they contained six imidazole molecules and no water, which is in agreement with the calculated composition of [Co(C_3_H_4_N_2_)_6_](NO_3_)_2_ and [Co(C_3_H_4_N_2_)_6_](ClO_4_)_2_ (Table 1).

**Figure 1:**
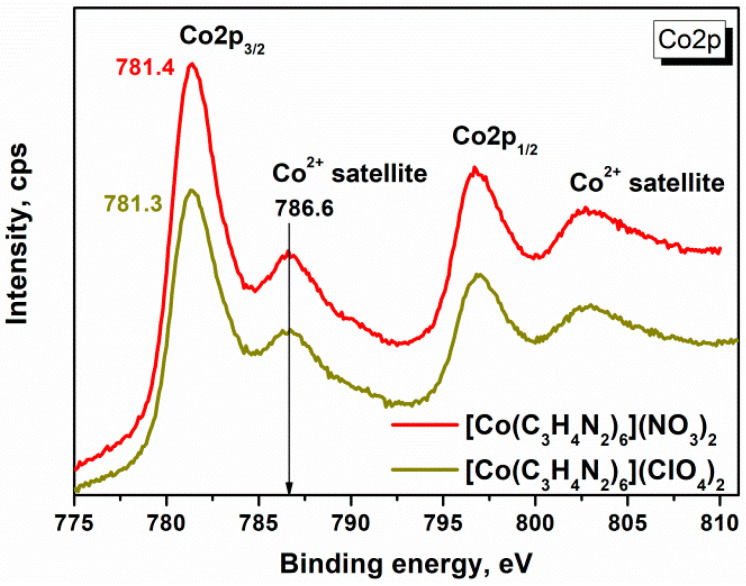
XPS spectra of Co2p for [Co(C_3_H_4_N_2_)_6_](NO_3_)_2_ and [Co(C_3_H_4_N_2_)_6_](ClO_4_)_2_ complexes.

The charge state of cobalt in the synthesized complexes with imidazole was determined using XPS. It can be seen from the XPS spectrum (Figure 1) that the binding energies of Co2p_3/2_ were 781.4 and 781.3 eV for Co(C_3_H_4_N_2_)_6_](NO_3_)_2_ and [Co(C_3_H_4_N_2_)_6_](ClO_4_)_2_, respectively. These values are very close to those for cobalt complexes, where the Co^2+^ ion is coordinated by the nitrogen of the imidazole ring [10,11]. In addition, there is a maximum of 786.6 eV, corresponding to the satellite of the ionic form of cobalt. The difference between the main peaks and the satellites is an important indicator of the oxidation state of the cobalt ion. A narrow separation of about 4–6 eV is a typical characteristic of Co^2+^, whereas a larger gap of about 9–10 eV is often found in Co^3+^ [12]. The separation between the main peak and the adjacent satellite peak was about 5 eV (Figure 1). Thus, despite the presence of anion oxidizers in the synthesized complexes, the oxidation of cobalt to Co^3+^ does not occur, as was observed in the synthesis of complexes with imidazole [13] and ethylenediamine [14] in aqueous solutions in the presence of atmospheric oxygen.

**Figure 2:**
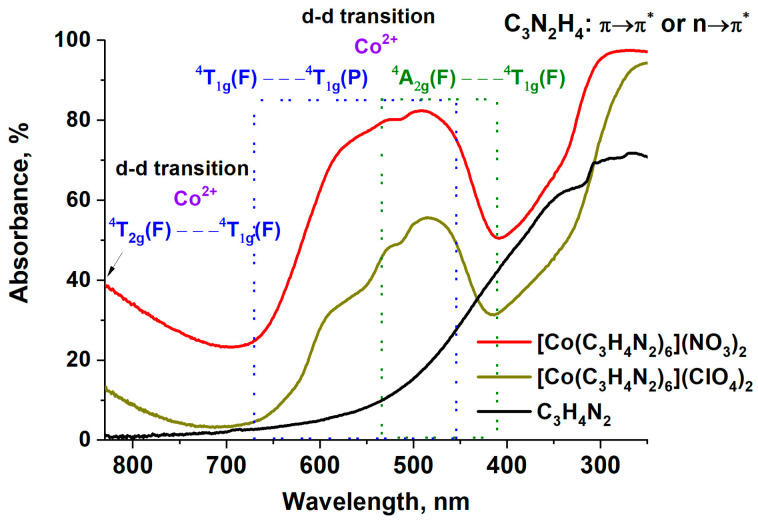
Diffuse reflectance UV–vis spectra for imidazole in [Co(C_3_H_4_N_2_)_6_](NO_3_)_2_ and [Co(C_3_H_4_N_2_)_6_](ClO_4_)_2_ complexes.

To determine the nature of the coordination between the central atom and the imidazole molecules, cobalt complexes were studied using diffuse reflectance UV–vis spectroscopy. The obtained spectra of the [Co(C_3_H_4_N_2_)_6_](NO_3_)_2_ and [Co(C_3_H_4_N_2_)_6_](ClO_4_)_2_ complexes (Figure 2) were almost identical. Hence, the type of coordination of the cation by the imidazole molecules is the same, despite the different nature of the anion.

A detailed analysis of the spectra (Figure 2) showed that two regions of intense absorption could be distinguished in the wavelength range from 700 to 250 nm. Absorption below 450 nm corresponds to π→π* transitions in imidazole. Note that the complexes are characterized by a higher absorption intensity than that of the original imidazole. It is known [15] that a weakening of the hypochromic effect is observed due to a decrease in imidazole molecule interactions between each other, i.e., the imidazole molecules become isolated upon the complex formation.

The second intense absorption at 600–500 nm (Figure 2) can be attributed to d-d transitions of Co^2+^ in high-spin octahedral complexes, with three transitions in the UV–vis spectra [16]:

υ_1_^4^T_2g_---^4^T_1g_ 950–1250 nm weak

υ_2_^4^A_2g_---^4^T_1g_ 420–520 nm weak (shoulder)

υ_3_^4^T_1g_(P)---^4^T_1g _ 450–600 nm strong

The multiplet structure of the absorption in the region of 600–500 nm is due to close energy values for the transitions ^4^T_1g_(P)---^4^T_1g_ and ^4^A_2g_---^4^T_1g_, where the latter appears as a shoulder, since the υ_3_/υ_2_ ratio ranges from 1.9 to 2.2 for octahedral Co^2+^ complexes with different ligands [17,18]. Summarizing the results of the UV–vis spectroscopy, we can conclude that the Co^2+^ cation is in an octahedral environment of imidazole molecules that are isolated from each other. The anion nature in the complex has a negligible effect on the coordination of the central atom with the ligand.

**Figure 3:**
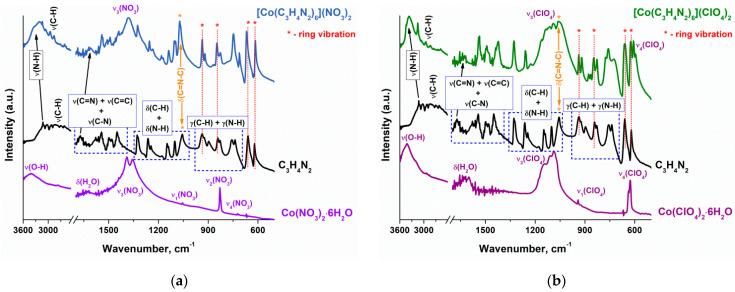
ATR FTIR data obtained for (**a**) [Co(C_3_H_4_N_2_)_6_](NO_3_)_2_ and (**b**) [Co(C_3_H_4_N_2_)_6_](ClO_4_)_2_ complexes.

The interaction between the cobalt ions and the imidazole molecules in the [Co(C_3_H_4_N_2_)_6_](NO_3_)_2_ and [Co(C_3_H_4_N_2_)_6_](ClO_4_)_2_ complexes was described based on ATR FTIR spectroscopy data. According to Figure 3, the absorption bands of the υ_3_(NO_3_) and υ_3_(ClO_4_) modes [19] do not change their position or intensity. So, it can be argued that the anions remain in the outer coordination sphere of cobalt, as in the original salts Co(NO_3_)_2_·6H_2_O and Co(ClO_4_)_2_·6H_2_O.

When forming the nearest cobalt environment, imidazole also does not undergo any structural rearrangements because the characteristic bands of the imidazole ring remain [20]. However, the absorption bands of the N-H stretching vibrations are shifted to a higher-frequency region (from 3100 to 3300 cm^−1^). As was demonstrated in [21,22] based on calculation methods, such changes can be explained by the breaking of hydrogen bonds between imidazole molecules. The reason of their formation is the presence of a weakly acidic >N-H group in the imidazole ring and a nitrogen atom (–N=) with a lone electron pair capable of proton attaching. As a result of the complex formation, hydrogen bonds are broken and the N-H group becomes isolated, which shortens the bond length.

The interaction of imidazole with cobalt cations is also confirmed by the spectrum changes in the region of C-N bond vibrations (Figure 3). Indeed, the absorption bands of C-N bond stretching vibrations shift from 1670 to 1610 and 1630 cm^−1^ for the [Co(C_3_H_4_N_2_)_6_](NO_3_)_2_ and [Co(C_3_H_4_N_2_)_6_](ClO_4_)_2_ complexes, respectively (Figure 3). This indicates an increase in the C-N bond length due to the redistribution of electron density from nitrogen to metal. At the same time, a noticeable increase in the band intensity at 1060 ± 10 cm^−1^ relative to the other bands of imidazole ring is observed. This absorption band is related to the deformation vibrations of the (C=N-C) bond [20]; therefore, it can be assumed that the intensification of vibrations is due to the weakening of the bond between nitrogen and carbon.

**Figure 4:**
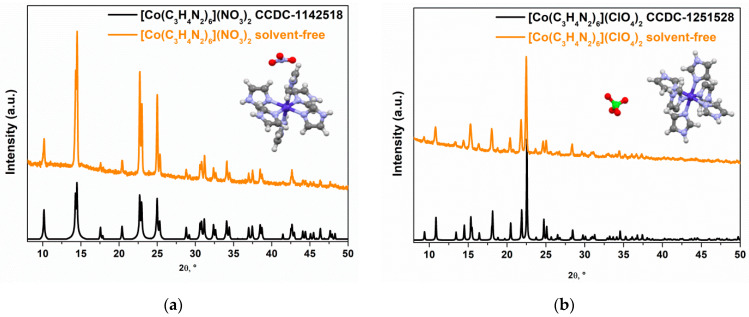
PXRD data obtained for (**a**) [Co(C_3_H_4_N_2_)_6_](NO_3_)_2_ and (**b**) [Co(C_3_H_4_N_2_)_6_](ClO_4_)_2_ complexes.

Final confirmation of the formation of [Co(C_3_H_4_N_2_)_6_](NO_3_)_2_ and [Co(C_3_H_4_N_2_)_6_](ClO_4_)_2_ complexes upon the addition of cobalt salts to the imidazole melt in a stoichiometric amount was obtained by the powder XRD method (Figure 4). It was found that all peaks in the XRD pattern of the complexes containing the coordination site of CoN_6_ matched the theoretical powder pattern of [Co(C_3_H_4_N_2_)_3_](NO_3_)_2_ and [Co(C_3_H_4_N_2_)_3_](ClO_4_)_2_ plotted from the CIF [23].

Summarizing the results of the study with several physicochemical methods, we can conclude that imidazole melting with cobalt nitrate or perchlorate produces complexes with a CoN_6_ coordination environment. In this case, the Co^2+^ ion interacts with the pyridine nitrogen atom, and the anions are located in the outer sphere of the cation. Thus, we have proposed in our work a novel solvent-free method for the synthesis of [Co(C_3_H_4_N_2_)_6_](NO_3_)_2_ and [Co(C_3_H_4_N_2_)_6_](ClO_4_)_2_ complexes.

### 2.2. Study of Thermal Decomposition of the Cobalt Complexes

**Figure 5:**
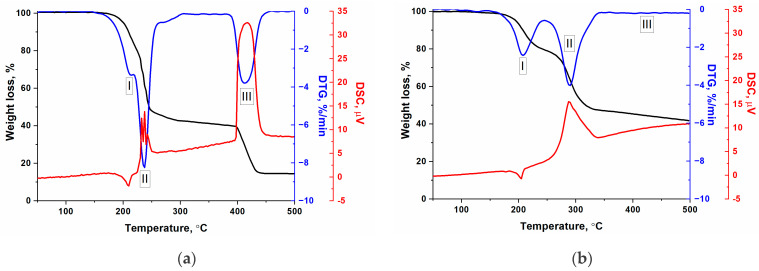
Thermal decomposition of (**a**) [Co(C_3_H_4_N_2_)_6_](NO_3_)_2_ and (**b**) [Co(C_3_H_4_N_2_)_6_](ClO_4_)_2_ complexes in air with a heating rate of 5 °C·min^−1^. I, II, and III—thermal decomposition stages.

The principle of operation of gas-generation compositions is based on the release of a large volume of low-molecular-weight gases due to the thermochemical transformations of their components. This section presents data on the features of the thermal decomposition of the cobalt complexes synthesized in imidazole melt. It was noted that there were no endothermic effects on DSC curves (Figure 5) caused by the melting of imidazole at 90 °C [24] or the decomposition of cobalt salts at 200–300 °C [25], which minimized the presence of reagent impurities in the sample.

According to the data obtained (Figure 5), [Co(C_3_H_4_N_2_)_6_](NO_3_)_2_ and [Co(C_3_H_4_N_2_)_6_](ClO_4_)_2_ complexes are thermally stable up to 150 and 170 °C, respectively. However, intense decomposition with a pronounced exothermic effect is observed above 200 °C. Based on DSC data, we calculated the critical temperature of thermal decomposition, where equilibrium is achieved between the heat loss of the sample and the exothermic effect of its thermochemical transformations. Hence, this parameter allows one to evaluate the thermal stability of substances, according to Xue’s [26] method. The obtained values for the [Co(C_3_H_4_N_2_)_6_](NO_3_)_2_ and [Co(C_3_H_4_N_2_)_6_](ClO_4_)_2_ complexes were 227 and 253 °C, respectively. Thus, the perchlorate complex is more thermostable, and it is also characterized by a lower specific heat of combustion, despite the higher oxygen content (Table 1).

**Figure 6:**
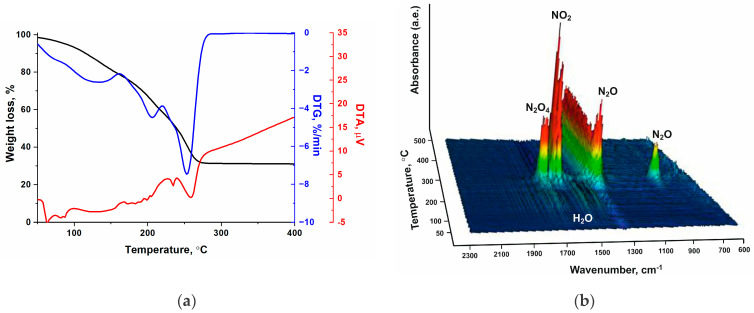
Thermal decomposition of Co(NO_3_)_2_·6H_2_O in air with a heating rate of 10 °C·min^−1^: (**a**) thermal analysis data obtained by TG and DTA; (**b**) TG-FTIR data of released gases.

In general, the thermal decomposition of [Co(C_3_H_4_N_2_)_6_](NO_3_)_2_ and [Co(C_3_H_4_N_2_)_6_](ClO_4_)_2_ complexes in the temperature range from 50 to 500 °C and at a heating rate of 5 °C/min proceeds through several stages by mass change, according to the change in the mass of the sample. In the case of the [Co(C_3_H_4_N_2_)_6_](NO_3_)_2_ complex, three stages of thermal decomposition can be distinguished, whereas for the [Co(C_3_H_4_N_2_)_6_](ClO_4_)_2_ complex, only two stages are clearly identified (Figure 5b). The third stage is weakly expressed due to the slow oxidation of the products and the incomplete decomposition of the ligand, probably due to the presence of chlorine. The first endothermic stage begins near 200 °C and reaches maximal mass loss at 210 ± 3 °C. As a result, the mass of the samples decreases by 12 ± 2%, which corresponds to the elimination of one imidazole molecule. Then, an additional mass loss of about 40% accompanied by the heat release is observed. Most likely, in this temperature region, the organic part of the complex is partially oxidized with both oxygen and nitrogen oxides, or chlorine formed during the decomposition of the nitrate anions (Figure 6) and perchlorate anions of the transition metals [27]:

M(ClO_4_)_2_ → MO + Cl_2_ + 3.5O_2_.

**Figure 7:**
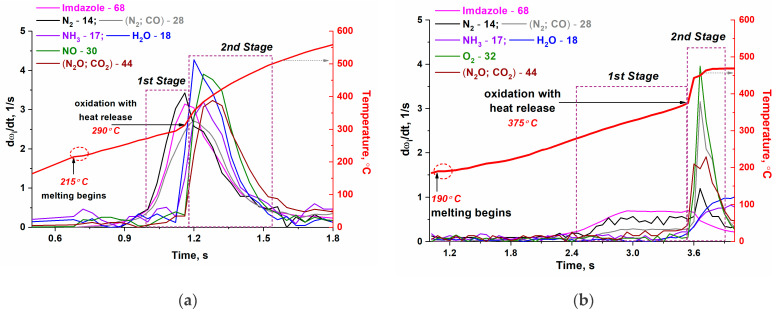
The composition of gaseous products during thermal decomposition of (**a**) [Co(C_3_H_4_N_2_)_6_](NO_3_)_2_ and (**b**) [Co(C_3_H_4_N_2_)_6_](ClO_4_)_2_ complexes in argon with a high rate of heat. Method of dynamic mass spectrometry with simultaneous temperature measurement. (dω_i_/dt—change in i-component fraction in the gas phase with time).

More detailed information on the composition of the released gases was obtained during the high-speed gasification of the complexes, close to real gas-generation conditions (Figure 7). It was found using dynamic mass spectrometry with simultaneous temperature measurement that no decomposition products of the complex are released during the endothermic stage, except for a small amount of water in the case of the [Co(C_3_H_4_N_2_)_6_](NO_3_)_2_ complex. Therefore, heat absorption is the result of melting, which begins at 215 and 190 °C for the [Co(C_3_H_4_N_2_)_6_](NO_3_)_2_ and [Co(C_3_H_4_N_2_)_6_](ClO_4_)_2_ complexes, respectively, under high-speed heating conditions.

The first decomposition products of the complexes are identified at temperatures above 270 °C, and we can distinguish (1) low-temperature and (2) high-temperature stages according to the composition and intensity of the products released. It should be noted that these stages are not completely identical to the stages determined from the thermal analysis data (Figure 5), since the heating rates are very high. In this case, the (1) low-temperature stage (Figure 7) includes stages I and II, determined from the results of the thermal analysis (Figure 5). At the (2) high-temperature stage, oxidative processes (stage III, thermal analysis data) occur, as evidenced by an abrupt increase in temperature.

At the (1) low-temperature stage, imidazole, N_2_, and/or CO are observed with an almost uniform temperature rise. The (2) high-temperature stage is characterized by intense heat release and a large amount of complete gasification products of the complex, mainly H_2_O, N_2_, CO, N_2_O, and CO_2_. In addition, in the case of the [Co(C_3_H_4_N_2_)_6_](NO_3_)_2_ complex, NO and NH_3_ are formed, but there is no oxygen, which is typical for the decomposition of a nickel complex of similar composition [9]. For the [Co(C_3_H_4_N_2_)_6_](ClO_4_)_2_ complex, the release of oxygen was observed at 450 °C, and HCl was a minor product of its thermal decomposition. It can be assumed from the obtained dynamic mass spectrometry data that all the oxygen released during the decomposition of the nitrate anion [28] is spent on the oxidation of the organic ligand. Therefore, the degree of gasification of the nitrate complex is significantly higher than that of the perchlorate complex (Figure 5). As a result, the total mass loss of the nitrate complex was 85.5%, and the remaining mass of the sample (14.5%) was very close to the value corresponding to the formation of Co_3_O_4_ (13.6%).

**Figure 8:**
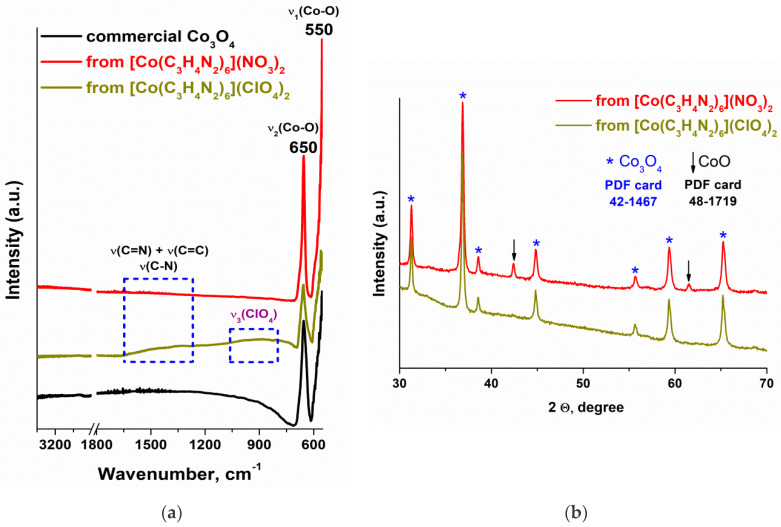
(**a**) ATR FTIR and (**b**) XRD data obtained after thermal treatment of [Co(C_3_H_4_N_2_)_6_](NO_3_)_2_ and [Co(C_3_H_4_N_2_)_6_](ClO_4_)_2_ complexes at 500 °C.

IR spectroscopy and X-ray diffraction methods confirmed the formation of cobalt oxides during the thermal decomposition of the [Co(C_3_H_4_N_2_)_6_](NO_3_)_2_ and [Co(C_3_H_4_N_2_)_6_](ClO_4_)_2_ complexes (Figure 8). Indeed, two absorption bands at ~550 (Co^3+^ vibration in the octahedral hole) and ~650 cm^−1^ (Co^2+^ vibration in the tetrahedral hole), characteristic of Co_3_O_4_ spinel [29], were observed in the IR spectra of the samples after heat treatment in air (Figure 8a). The main reflections in the diffraction patterns of the thermal decomposition products of the [Co(C_3_H_4_N_2_)_6_](NO_3_)_2_ and [Co(C_3_H_4_N_2_)_6_](ClO_4_)_2_ complexes also belong to this phase (Figure 8b).

It should be noted that the gasification of the [Co(C_3_H_4_N_2_)_6_](NO_3_)_2_ and [Co(C_3_H_4_N_2_)_6_](ClO_4_)_2_ complexes is a nonequilibrium process, and along with the formation of Co_3_O_4_ and the oxidation of the organic ligand, reactions, which form the additional phases, occur in the samples. The analysis of the XRD data (Figure 8b) showed that it is not just Co_3_O_4_ that is formed during the thermal decomposition of the Co(C_3_H_4_N_2_)_6_](NO_3_)_2_ complex; the CoO phase also occurs, which has characteristic reflections at 42 and 61°. Note that they are absent in the diffraction patterns of the solid gasification products of the [Co(C_3_H_4_N_2_)_6_](ClO_4_)_2_ complex. However, the low degree of gasification of this complex (mass loss of about 58%) suggests the presence of amorphous impurities in the solid thermal decomposition products. Indeed, two regions of structureless absorption can be additionally distinguished in its IR spectrum at 1300–1600 cm^−1^ and 800–1100 cm^−1^ (Figure 8a). Higher-frequency absorption relates to the stretching vibrations of the C=C, C=N, and C-N bonds in carbon materials, including those containing nitrogen [30,31]. The reason for absorption at 800–1100 cm^−1^ can be both the presence of perchlorate anions in the sample [32], and the deformation and rocking vibrations of the C-H and N-H bonds [33]. However, there are no absorption bands in the IR spectrum at 3600–3000 cm^−1^ (Figure 8a) related to the stretching vibrations of the C-H and N-H bonds. Therefore, absorption at 800–1100 cm^−1^ is more likely due to the asymmetric stretching vibrations in the ClO_4_^−^ ion, and absorption at 1300–1600 cm^−1^ is associated with the presence of amorphous carbon doped with nitrogen resulting from the carbonization of the organic ligand of the [Co(C_3_H_4_N_2_)_6_](ClO_4_)_2_ complex.

### 2.3. Evaluation of Activation Energy for Thermal Decomposition of Cobalt Complexes

**Figure 9:**
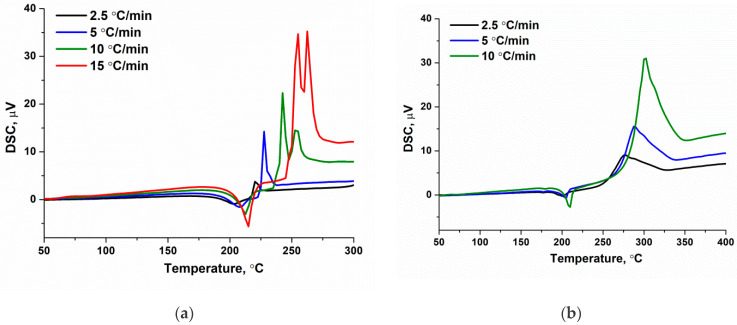
The DSC curves of (**a**) [Co(C_3_H_4_N_2_)_6_](NO_3_)_2_ and (**b**) [Co(C_3_H_4_N_2_)_6_](ClO_4_)_2_ complexes in air with heating rates of β = 2.5, 5, and 10 °C·min^−1^; 15 °C·min^−1^ was only used for [Co(C_3_H_4_N_2_)_6_](NO_3_)_2_.

The evaluation of the activation energy (E_a_) for the endothermic and exothermic stages of [Co(C_3_H_4_N_2_)_6]_(NO_3_)_2_ and [Co(C_3_H_4_N_2_)_6_](ClO_4_)_2_ complex decomposition was performed using thermal analysis data by varying the heating rate β between 2.5, 5, and 10 °C·min^−1^. The rate of 15 °C·min^−1^ was only used only for [Co(C_3_H_4_N_2_)_6_](NO_3_)_2_. The Kissinger model was chosen for the analysis of kinetic data as it does not require knowledge of the process mechanism [34], but thermal effects should occur at similar conversion values. For this purpose, we identified the extreme temperatures for the endothermic (conversion 10 ± 2%) and exothermic (conversion 40 ± 3%) stages of [Co(C_3_H_4_N_2_)_6]_(NO_3_)_2_ and [Co(C_3_H_4_N_2_)_6_](ClO_4_)_2_ complex decomposition at all heating rates (Figure 9). From the obtained values, the activation energies for the first two stages were acquired (Table 2). As in the case of the critical temperature of thermal explosion, the [Co(C_3_H_4_N_2_)_6_](ClO_4_)_2_ complex is characterized by a higher activation energy than [Co(C_3_H_4_N_2_)_6]_(NO_3_)_2_.

Thus, the main stages of thermal decomposition of the [Co(C_3_H_4_N_2_)_6_](NO_3_)_2_ and [Co(C_3_H_4_N_2_)_6_](ClO_4_)_2_ complexes were analyzed, allowing us to evaluate their activation energies. The obtained results indicate the higher thermal stability of the perchlorate cobalt complex, characterized by a high critical temperature of thermal explosion and a low degree of gasification. Hence, this explains the low degree of its gasification and the higher content of carbon- and chlorine-containing impurities in the products of thermal decomposition.

## 3. Materials and Methods

### 3.1. Materials

For the synthesis of complex compounds, the following commercially available chemical reagents were used: Co(NO_3_)_2_·6H_2_O (GOST 4528-78, 97%), 2CoCO_3_·3Co(OH)_2_·H_2_O (GOST 5407-78, 98%), C_3_H_4_N_2_ (TU 6-09-08-1314-78, 99%), HClO_4_ (TU 6-09-2878-84, 60%), Co_3_O_4_ (GOST 4467-79, 99.0%).

### 3.2. Synthesis of Complex Compounds [Co(C_3_H_4_N_2_)_6_](NO_3_)_2_ and [Co(C_3_H_4_N_2_)_6_](ClO_4_)_2_

#### 3.2.1. Synthesis of Co(ClO_4_)_2_·6H_2_O

First, 0.04 moles of HClO_4_ was dripped to 0.008 moles of 2CoCO_3_·3Co(OH)_2_·H_2_O and stirred thoroughly. Then, a small amount of distilled water (~5 mL) was added, and the solution was evaporated (T_furnace_ = 90 °C) and filtered on a Schott filter using a double-necked flask. The resulting pink precipitate was dried in the air. The yield was 89%, and the purity was 98.7% (based on cobalt content).

#### 3.2.2. Solvent-Free Synthesis of [Co(C_3_H_4_N_2_)_6_](NO_3_)_2_ and [Co(C_3_H_4_N_2_)_6_](ClO_4_)_2_

The synthesis was performed within a ceramic crucible on the heated surface of an IKA-C-Mag HS4 tile (IKA, Staufen, Germany) at a predetermined temperature of 120 °C. First, 0.01 moles of cobalt salts ((Co(NO_3_)_2_·6H_2_O or Co(ClO_4_)_2_·6H_2_O)) was added to the melted imidazole C_3_H_4_N_2_ (0.06 mole, melting point = 89–91 °C), and an active interaction between the reagents was observed with the formation of blue humid powder that quickly dried and became pink-violet while being stirred. Afterwards, the obtained complex compound was dried in a desiccator under P_2_O_5_ and in a vacuum centrifuge (30 °C, 4 h). The yield was 97 ± 1%.

### 3.3. Characterization of Complex Compounds and Solid Products of Combustion

The Co content was found using inductively coupled plasma atomic emission spectrometry, employing an Optima 4300 DV (PerkinElmer, Waltham, MA, USA).

The carbon, hydrogen, and nitrogen contents were calculated with the use of an automatic CHNS-analyzer EURO EA 3000 (Euro Vector S.p.A., Castellanza, Italy). The samples (0.5–2 mg) were burned in a vertical reactor in a dynamic regime at 1050 °C in the flow of a helium–oxygen mixture.

The oxygen balance of the complexes was calculated using the following equation:

(1) $$Oxygen\;balance\;\left(\%\right)=\frac{-1600}{Molar\;mass}\cdot \left(2C+\frac{H}{2}+M-O\right),$$

where C, H, and O are the number of carbon, hydrogen, and oxygen atoms per molecule, respectively. M is $\frac{4}{3}$, assuming that the main product of cobalt oxidation is Co_3_O_4_.

The heat of the combustion of the complexes was determined in a Semimicro Calorimeter Parr 6725 with a Calorimetric Thermometer 6772 (Parr, Moline, IL, USA).

Diffuse reflectance UV–vis spectra were recorded in air at room temperature on a Varian Cary 100 instrument (Agilent, Santa Clara, CA, USA) equipped with a standard diffuse reflectance attachment. Samples in the form of powder were placed in the cell equipped with a quartz window.

The infrared spectra were obtained by attenuated total reflection infrared spectroscopy (ATR FTIR) employing an Agilent Cary 600 spectrometer (Agilent Technologies, Santa Clara, CA, USA) with a Gladi ATR attachment (PIKE Technologies, Madison, WI, USA) in the range of wavenumbers 250–4000 cm^−1^ without a preliminary sample preparation.

The powder X-ray diffraction (XRD) patterns of the synthesized complexes and the solid products of their combustion were obtained in the 2θ range from 10 to 70° with a step of 0.02° and speed of 2°/min using an ARL X’tra (Thermo, Ecublens, Switzerland) diffractometer equipped with a linear detector Mythen2R 1D (Dectris, Baden, Switzerland). CuK_α_ radiation (λ = 1.5418 Å) was used. The composition of the gasification products was identified by the Rietveld method [35]. The average sizes of the coherent scattering regions (CSRs) were calculated using the Scherer formula by reflexes 311 for Co_3_O_4_ and 200 for CoO. The diffraction profile was described using the Fityk program, and the Pseudo Voigt function was used.

Crystallographic data (excluding structure factors) for the structures in this paper have been deposited at the Cambridge Crystallographic Data Centre, CCDC, 12 Union Road, Cambridge CB21EZ, UK. Copies of the data can be obtained free of charge by quoting the depository numbers CCDC-1142518 for [Co(C_3_H_4_N_2_)_6_](NO_3_)_2_ and CCDC-1251528 for [Co(C_3_H_4_N_2_)_6_](ClO_4_)_2_ [23].

The XPS spectra of the samples were recorded with an accuracy of ±0.1 eV and a depth of about 5 nm using a SPECS photoelectron spectrometer with a PHOIBOS-150-MCD-9 hemispheric analyzer and a FOCUS-500 monochromator (SPECS Surface Nano Analysis GmbH, Berlin, Germany) (AlK_α_ radiation, 150 W, hν = 1486.74 eV). The binding energy (BE) scale of the spectrometer was pre-calibrated using Au4f_7/2_ (84.0 eV) and Cu2p_3/2_ (932.6 eV) core-level peaks. The samples were supported on a conducting scotch. The sample charge was taken into account using C1s lines (284.8 eV). The individual spectra of elements allowed us to determine their electronic state and to calculate the ratios of different forms of cobalt present on the catalyst surface, taking into account the element sensitivity coefficients [36].

A Netzsch STA 449 C Jupiter instrument equipped with a DSC/TG holder (NETZSCH, Selb, Germany) was employed to study the thermal decomposition of [Co(C_3_H_4_N_2_)_6_](NO_3_)_2_ and [Co(C_3_H_4_N_2_)_6_](ClO_4_)_2_ in the temperature range of 30–500 °C under a flow of air (30 mL·min^−1^). The heating rates were 2.5, 5, 10, and 15 °C·min^−1^ for [Co(C_3_H_4_N_2_)_6_](NO_3_)_2_ and 2.5, 5, and 10 °C·min^−1^ for [Co(C_3_H_4_N_2_)_6_](ClO_4_)_2_. The gas composition was determined using an Agilent Cary 600 FTIR-spectrometer (Agilent Technologies Australia, Melbourne, Australia). The internal volume of the gas cell was 100 cm^3^ and the optical path length was 10 cm.

The composition of the gaseous products from the combustion was analyzed by the dynamic mass spectral thermal analysis (DMSTA) method, using a time-of-flight mass spectrometer with a molecular beam sampling system MSCh-4 (Plant Of Scientific Instrumentation, Sumy, USSR) under a flow of Ar (5 mL·min^−1^). The average heating rate ranged from 100 to 245 °C·s^−1^. The sample weight was 1–5 mg. The delay between measurements was 0.04 s. The identification of mass spectral signals was carried out using the mass spectra of individual substances from the NIST database [37].

### 3.4. Study of the Thermal Properties of [Co(C_3_H_4_N_2_)_6_](NO_3_)_2_ and [Co(C_3_H_4_N_2_)_6_](ClO_4_)_2_

The kinetic parameters of the thermal decomposition of [Co(C_3_H_4_N_2_)_6_](NO_3_)_2_ and [Co(C_3_H_4_N_2_)_6_](ClO_4_)_2_ were calculated using the Kissinger method [34] based on the DSC data for different heating rates. The Kissinger equation is used to find the activation energy of non-isothermal processes without the assumptions of kinetic models:

(2) $$ln\left(\frac{\beta }{T_{m}^{2}}\right)=-\frac{E}{RT_{m}}+\ln\left(\frac{AR}{E}\right),\;$$

where β is the heating rate; T_m_ is the temperature of the peak of the reaction rate; E is the activation energy of the reaction; R is the universal gas constant; and A is the pre-exponential factor.

In addition, a comparison of the thermal stability of the synthesized complexes was completed based on the critical temperature of thermal decomposition using the method provided by Xue et al.

(3) $$T_{b}=T_{0}+\frac{\partial T_{e}}{\partial \left(ln\beta \right)},$$

where T_e_ is the onset temperature as a function of β, and T_0_ is the onset temperature extrapolated to β = 0 using the 3rd-order polynomial fit [26].

## 4. Conclusions

A solvent-free method for the synthesis of complex compounds of imidazole with cobalt ([Co(C_3_H_4_N_2_)_6_](NO_3_)_2_ and [Co(C_3_H_4_N_2_)_6_](ClO_4_)_2_) was proposed. Elemental analysis, XPS, IR spectroscopy, and XRD confirmed the formation of the complexes in the CoN_6_ coordination environment during the interaction of cobalt salts with imidazole in the melt. In this case, Co^2+^ ions are coordinated by pyridine nitrogen atoms, and the anions are located in the outer sphere of the cation.

The study of the thermal decomposition of the [Co(C_3_H_4_N_2_)_6_](NO_3_)_2_ and [Co(C_3_H_4_N_2_)_6_](ClO_4_)_2_ complexes showed that they are thermally stable up to 150 and 170 °C, respectively. When the critical temperature of thermal decomposition is reached, their fast gasification is observed with the release of a large amount of heat. In this case, the organic ligand in the [Co(C_3_H_4_N_2_)_6_](NO_3_)_2_ complex undergoes almost complete gasification, probably due to the oxygen formed during the thermal decomposition of the nitrate anion. That is why oxygen was not identified with the dynamic mass spectrometry in the gaseous products of the complex thermal decomposition, as is typical for nickel complexes of a similar composition [9]. As a result, Co_3_O_4_ with a slight admixture of CoO is formed.

The more thermally stable [Co(C_3_H_4_N_2_)_6_](ClO_4_)_2_ complex is characterized by a lower degree of gasification (about 58%) due to the carbonization of the ligand with the formation of amorphous carbon containing nitrogen. Probably, the main reasons for this are (I) the higher thermal stability of the perchlorate anion compared to the nitrate one [27]; (II) the weaker oxidizing properties of chlorine than those of oxygen and nitrogen oxides formed during the decomposition of anions; (III) the higher values of activation energy (Table 2) and the critical temperature of thermal explosion; (IV) less heat being released during the oxidative gasification of the complex (Table 1).

Summarizing the research performed, we can conclude that the [Co(C_3_H_4_N_2_)_6_](NO_3_)_2_ complex is a promising component for gas-generation compounds, such as airbags. Moreover, its synthesis can be carried out without the use of solvents, in accordance with the principles of “Green Chemistry”.

## Figures and Tables

**Table 1:** Characteristics of the synthesized nickel complexes.

Complex, Molar Mass	Composition, %		Gross Formula	Oxygen Balance	Heat of Combustion
	Calculated	Found			
[Co(C_3_H_4_N_2_)_6_](NO_3_)_2_ CoC_18_H_24_N_14_O_6_ 591 g/mol	Co—10.0 C—36.6 H—4.1 N—33.2 O—16.2	Co—10.5 C—36.1 H—3.9 N—32.7 Other («O») ^1^—16.8	CoC_16.9_H_21.9_N_13.1_«O»_5.9_	−117%	17.6
[Co(C_3_H_4_N_2_)_6_](ClO_4_)_2_ CoC_18_H_24_Cl_2_N_12_O_8_ 666 g/mol	Co—8.8 C—32.4 H—3.6 N—25.2 Cl—10.6 O—19.2	Co—8.9 C—32.5 H—3.6 N—25.2 Cl—10.6 Other («O») ^1^—19.2	CoC_17.9_H_23.8_N_11.9_«O»_7.9_Cl_2_	−112%	14.7

^1^ Supposedly oxygen.

**Table 2:** Activation energy of different stages of thermal decomposition acquired by Kissinger method and the critical temperature of thermal explosion of [Co(C_3_H_4_N_2_)_6_](NO_3_)_2_ and [Co(C_3_H_4_N_2_)_6_](ClO_4_)_2_.

Complex	Stage 1 (Endothermic)			Stage 2 (Exothermic)		
	Heat Rate, °C/min	Temperature, °C	Activation Energy, kJ/mol	Heat Rate, °C/min	Temperature, °C	Activation Energy, kJ/mol
[Co(C_3_H_4_N_2_)_6_](NO_3_)_2_	2.5	203	275 ± 44	2.5	220	100 ± 13
	5	210		5	228	
	10	213		10	243	
	15	215		15	255	
[Co(C_3_H_4_N_2_)_6_](ClO_4_)_2_	2.5	200	283 ± 17	2.5	276	136 ± 5
	5	204		5	289	
	10	209		10	301	

## Data Availability

Data are contained within the article.

## References

[1] Muhammed A., Sreelakshmi P., Praseeda P.N., Jishnu, Shan S. (2021). A review on gas-generators for application in exploding fire extinguisher balls. IOP Conf. Ser. Mater. Sci. Eng..

[2] Barsukov V., Goldaev S., Basalaev S. (2019). Applying of open gas generators in solid fuel for rising underwater objects. AIP Conf. Proc..

[3] Neutz J., König A., Knauss H., Jordan S., Rödiger T., Smorodsky B., Blümcke E.W. (2009). Mass Flow Discharge and Total Temperature Characterization of a Pyrotechnic Gas Generator Formulation for Airbag Systems. Propellants Explos. Pyrotech..

[4] Varisova R.R. (2022). Technologies for the development of high-viscosity oil fields. J. Phys. Conf. Ser..

[5] Netskina O.V., Dmitruk K.A., Mazina O.I., Paletsky A.A., Mukha S.A., Prosvirin I.P., Pochtar A.A., Bulavchenko O.A., Shmakov A.G., Veselovskaya J.V. (2023). CO_2_ methanation: Solvent-Free Synthesis of Nickel-Containing Catalysts from Complexes with Ethylenediamine. Materials.

[6] Tella A.C., Eke U.B., Owalude S.O. (2016). Solvent-free mechanochemical synthesis and X-ray studies of Cu(II) and Ni(II) complexes of 5-(3,4,5-Trimethoxybenzyl)pyrimidine-2,4-diamine (Trimethoprim) in a ball-mill. J. Saudi Chem. Soc..

[7] Netskina O., Mukha S., Veselovskaya J., Bolotov V., Komova O., Ishchenko A., Bulavchenko O., Prosvirin I., Pochtar A., Rogov V. (2021). CO_2_ Methanation: Nickel-Alumina Catalyst Prepared by Solid-State Combustion. Materials.

[8] Netskina O.V., Mukha S.A., Dmitruk K.A., Ishchenko A.V., Bulavchenko O.A., Pochtar A.A., Suknev A.P., Komova O.V. (2022). Solvent-Free Method for Nanoparticles Synthesis by Solid-State Combustion Using Tetra(Imidazole)Copper(II) Nitrate. Inorganics.

[9] Netskina O.V., Dmitruk K.A., Paletsky A.A., Mukha S.A., Pochtar A.A., Bulavchenko O.A., Prosvirin I.P., Shmakov A.G., Ozerova A.M., Veselovskaya J.V. (2022). Solvent-Free Synthesis of Nickel Nanoparticles as Catalysts for CO_2_ hydrogenation to Methane. Catalysts.

[10] Chai L.-Q., Zhou L., Zhang K.-Y., Zhang H.-S. (2018). Structural characterizations, spectroscopic, electrochemical properties, and antibacterial activities of copper (II) and cobalt (II) complexes containing imidazole ring. Appl. Organomet. Chem..

[11] Roncaroli F., Dal Molin E.S., Viva F.A., Bruno M.M., Halac E.B. (2015). Cobalt and Iron Complexes with N-heterocyclic Ligands as Pyrolysis Precursors for Oxygen Reduction Catalysts. Electrochim. Acta.

[12] Barreca D., Massignan C., Daolio S., Fabrizio M., Piccirillo C., Armelao L., Tondello E. (2001). Composition and microstructure of cobalt oxide thin films obtained from a novel cobalt (II) precursor by chemical vapor deposition. Chem. Mater..

[13] Pająk M., Woźniczka M., Vogt A., Kufelnicki A. (2017). Reversible uptake of molecular oxygen by heteroligand Co(II)–l-α-amino acid–imidazole systems: Equilibrium models at full mass balance. Chem. Cent. J..

[14] Norkus E., Vaškelis A., Grigucevičienė A., Rozovskis G., Reklaitis J., Norkus P. (2001). Oxidation of cobalt(II) with air oxygen in aqueous ethylenediamine solutions. Transit. Met. Chem..

[15] Peral F., Gallego E. (1997). Self-association of imidazole and its methyl derivatives in aqueous solution. A study by ultraviolet spectroscopy. J. Mol. Struct..

[16] Reimann C.W. (1966). Electron Absorption Spectrum of Cobalt (II)–Doped Trisphenanthrolinezinc Nitrate Dihydrate. J. Res. Natl. Bur. Standards. Sect. A Phys. Chem..

[17] Battistuzzi R. (1985). Cobalt(II) perchlorate, tetrafluoroborate, nitrate and sulphate complexes with 4,6-dimethylpyrimidine-2(1H)-thione. Polyhedron.

[18] Solomon E., Lever I.A.B.P. (1999). Inorganic Electronic Structure and Spectroscopy. Volume II. Applications and Case Studies.

[19] Hodgson J.B., Percy G.C., Thornton D.A. (1980). The IR spectra of imidazole complexes of 1st transition series metal(II) nitrates and perchlorates. J. Mol. Struct..

[20] Madanagopal A., Periandy S., Gayathri P., Ramalingam S., Xavier S., Ivanov V.K. (2017). Spectroscopic and computational investigation of the structure and pharmacological activity of 1-benzylimidazole. J. Taibah Univ. Sci..

[21] Lee S., Lee S.J., Ahn A., Kim Y., Min A., Choi M.Y., Miller R.E. (2011). Infrared Spectroscopy of Imidazole Trimer in Helium Nanodroplets: Free NH Stretch Mode. Bull. Korean Chem. Soc..

[22] Tikhonov D.S., Scutelnic V., Sharapa D.I., Krotova A.A., Dmitrieva A.V., Obenchain D.A., Schnell M. (2023). Structures of the (Imidazole)_n_H+ … Ar (n = 1, 2, 3) complexes determined from IR spectroscopy and quantum chemical calculations. Struct. Chem..

[23] CCDC Advancing Structural Science. https://www.ccdc.cam.ac.uk/.

[24] Grimmett M.R. (1970). Advances in Imidazole Chemistry. Adv. Heterocycl. Chem..

[25] Małecka B., Łącz A., Drożdż E., Małecki A. (2015). Thermal decomposition of d-metal nitrates supported on alumina. J. Therm. Anal. Calorim..

[26] Xue L., Zhao F.Q., Hu R.Z., Gao H.X. (2010). A Simple Method to Estimate the Critical Temperature of Thermal Explosion for Energetic Materials Using Nonisothermal DSC. J. Energ. Mater..

[27] Stern K.H. (1974). High temperature properties and decomposition of inorganic salts. Part 4. Oxy-Salts of the halogens. J. Phys. Chem. Ref. Data.

[28] Małecki A., Gajerski R., Łabuś S., Prochowska-Klisch B., Wojciechowski K.T. (2000). Mechanism of Thermal Decomposition of d-metals Nitrates Hydrates. J. Therm. Anal. Calorim..

[29] Manouchehri I., Kameli P., Salamati H. (2011). Facile Synthesis of Co_3_O_4_/CoO Nanoparticles by thermal treatment of ball-milled precursors. J. Supercond. Nov. Magn..

[30] Rodil S.E. (2005). Infrared spectra of amorphous carbon based materials. Diam. Relat. Mater..

[31] Das S.C., Majumdar A., Hippler R. (2014). Electronic and chemical property of amorphous carbon, hydrocarbon, hydrogenated/hydrogen free carbon nitride: Spectroscopic. Int. J. Innov. Sci. Res..

[32] Gowda N.M.N., Naikar S.B., Reddy G.K.N. (1984). Perchlorate ion complex. Adv. Inorg. Chem..

[33] Barroso-Bogeat A., Alexandre-Franco M., Fernández-González C., Gómez-Serrano V. (2014). FT-IR analysis of pyrone and chromene structures in activated carbon. Energy Fuels.

[34] Kissinger H.E. (1957). Reaction Kinetics in Differential Thermal Analysis. Anal. Chem..

[35] Rietveld H.M. (1969). A profile refinement method for nuclear and magnetic structures. J. Appl. Crystallogr..

[36] Scofield J.H. (1976). Hartree-Slater subshell photoionization cross-sections at 1254 and 1487 eV. J. Electron Spectrosc. Relat. Phenom..

[37] NIST Chemistry WebBook. https://webbook.nist.gov/chemistry/.

